# Multivariate Control of Effective Cobalt Doping in Tungsten Disulfide for Highly Efficient Hydrogen Evolution Reaction

**DOI:** 10.1038/s41598-018-37598-0

**Published:** 2019-02-04

**Authors:** Liyan Zhou, Shancheng Yan, Haizeng Song, Han Wu, Yi Shi

**Affiliations:** 1 Collaborative Innovation Center of Advanced Microstructures, Nanjing University, Nanjing, 210093 P. R. China; 2 School of Geography and Biological Information, Nanjing University of Posts and Telecommunications, Nanjing, 210023 P. R. China; 3 National Laboratory of Solid State Microstructures, School of Electronic Science and Engineering, Nanjing University, Nanjing, 210093 P. R. China

## Abstract

Tungsten Disulfide (WS_2_) is considered to be a promising Hydrogen Evolution Reaction (HER) catalyst to replace noble metals (such as Pt and Pd). However, progress in WS_2_ research has been impeded by the inertness of the in-plane atoms during HER. Although it is known that microstructure and defects strongly affect the electrocatalytic performance of catalysts, the understanding of such related catalytic origin still remains a challenge. Here, we combined a one-pot synthesis method with wet chemical etching to realize controlled cobalt doping and tunable morphology in WS_2_. The etched products, which composed of porous WS_2_, CoS_2_ and a spot of WO_x_, show a low overpotential and small Tafel slope in 0.5 M H_2_SO_4_ solution. The overpotential could be optimized to −134 mV (at 10 mA/cm^2^) with a Tafel slope of 76 mV/dec at high loadings (5.1 mg/cm^2^). Under N_2_ adsorption analysis, the treated WS_2_ sample shows an increase in macropore (>50 nm) distributions, which may explain the increase inefficiency of HER activity. We applied electron holography to analyze the catalytic origin and found a low surface electrostatic potential in Co-doped region. This work may provide further understanding of the HER mechanism at the nanometer scale, and open up new avenues for designing catalysts based on other transition metal dichalcogenides for highly efficient HER.

## Introduction

Hydrogen has been considered as a promising clean energy source to remedy the shortage of fossil fuel supply and pollution problems. As such, emerging non-noble catalysts for water splitting are in urgent need. Tungsten Disulfide (WS_2_), one of the two-dimensional transition metal dichalcogenides (TMDCs), has attracted much attention in the catalysis field due to the presence of active sites located along the edges^1–4^, and is expected to replace rare and expensive noble metal catalysts. However, apart from the edges, the majority of the basal surfaces are catalytically inert, which is the main limitation of bare TMDCs compared with noble metal catalysts. The design of the microstructures of such catalyst microstructures thus becomes important, the key directions being the exploration of the influence of pore size distributions, and even electrostatic potential distribution at the surface. There are several approaches reported to overcome the above-mentioned limitation and to enhance the catalytic performance of TMDCs. First, heteroatom doping can significantly modulate the catalytic activity of in-plane TMDC atoms; Deng *et al*. has systematically reported correlations between dopants and the catalytic activity of in-plane atoms of 2D MoS_2_^5^. Second, multiscale structures may help to promote the liquid-to-gas electrochemical conversion at the interface of catalysts. These mesoporous foams^6^, arrays^7,8^, nanodots^9,10^, and other nanostructures^11–13^ are commonly employed in catalytic researches. Especially for TMDCs, mesopores help to increase the number of edges, thus increasing the number of active sites. Third, high-conducting carbon-based materials including carbon nanotubes^14,15^, graphene^16–18^ and carbon cloth^19,20^ have been mixed with WS_2_ to improve its electrochemical properties. In addition, WS_2_ or MoS_2_ in the metallic 1T phase contains a higher density of exposed active sites, better conductivity and catalytic performance^21,22^, which can be obtained by chemical exfoliation from sample in the semiconducting 2H phase^23^. However, clear understanding of the effect of microstructure design on catalytic performance is still limited.

Herein, we combined pore size distribution analysis and electron holography for the first time to solve the above issue. A specific catalyst composed of Cobalt-doped and surface-etched WS_2_ was synthesized, showing efficient HER activity. We expect that Cobalt-doping will activate the inert in-plane atoms of WS_2_. Cobalt (Co) is demonstrated to be a good candidate to tune the free energy of hydrogen adsorption of WS_2_ and some other catalyst candidates^19,24^. Etching by H_2_O_2_ treatment with ultrasonication was applied to modify the morphology of WS_2_^25^, and also remove the redundant self-nucleated CoS_2_. The optimized sample displayed efficient HER performance, with an overpotential of −134 mV with a small Tafel slope of 76 mV/dec at a current density of 10 mA/cm^2^. The dependence of HER performance on sample microstructures was discussed; the nitrogen absorption test showed a better activity along with increasing macropore (>50 nm) distributions. An electron hologram has been taken to study the surface electrostatic potential, especially in Co-doped regions, bringing a new viewpoint into HER activity.

## Experimental

### Materials

All chemicals used in this work are of analytical grade and applied as received without further purification. Tungsten hexachloride (WCl_6_, 99.9%), thioacetamide (TAA, 99%), N-methylpyrrolidone (NMP, 99%) and Nafion solution (5%) were purchased from Sigma-Aldrich. Cobalt nitrate hexahydrate (Co(NO_3_)_2_·6H_2_O, 98.5%), and sulfuric acid (H_2_SO_4_, 98%) were supplied from Sinopharm Chemical Reagent Co. Ltd. Hydrogen peroxide (H_2_O_2_, 30%) was purchased from Nanjing Chemical Reagent Co. Ltd.

### Synthesis

In a typical synthesis of Co-doped WS_2_ (Co-WS_2_), WCl_6_ (0.8923 g), Co(NO_3_)_2_·6H_2_O (0.6341 g) and TAA (1.6904 g) were slowly added to deionized (DI) water (30 mL) and stirred at room temperature for 1 h. The solution was then transferred to a polyphenylene (PPL) reaction kettle and maintained at 265 °C for 24 h. The products were cooled to room temperature, centrifuged, washed several times with DI water and ethyl alcohol, and dried at 60 °C. WS_2_ was synthesized without Co(NO_3_)_2_·6H_2_O as a control.

For the preparation of H_2_O_2_-treated Co-WS_2_, 100 mg of Co-WS_2_ sample was dispersed in 20 mL of NMP with 2.5 vol% of H_2_O_2_ and then sonicated for 2 h. The products were centrifuged at 8000 rpm, washed several times, and dried at 60 °C. For comparation, Co-WS_2_ treated by H_2_O_2_ of different concentrations were prepared.

The products were annealed at 450 °C for 4 h under an Ar atmosphere before HER tests. We also studied the influence of different annealing conditions and passivation by 4-nitrobenzene-diazonium (4-NBD) on HER capability and stability, which can be seen in Supplementary Material (Fig. S1).

### Characterization

Field emission scanning electron microscopy (FE-SEM; JSM-7000F) was used to investigate the morphology of the samples. Transmission electron microscopy (TEM) and electron holography observations were obtained using a JEOL model JEM2100 instrument at an accelerating voltage of 200 kV. Energy dispersive spectrometer (EDS; inca x-stream 034A0) was used to confirm the stoichiometry of samples. The crystal phase properties of the samples were analyzed with a Bruker D8 Advance X-ray diffractometer (XRD) using Ni-filtered Cu Kα radiation at 40 kV and 40 mA at 2θ ranging from 10° to 70° with a scan rate of 0.02° per second. Raman spectra were obtained on a Raman spectrometer (LabRam HR800) excited by the 514.5 nm line of an Ar+ laser under 5 mW. X-ray photoelectron spectroscopy (XPS) analysis (PHI5000 Versaprobe) was used to determine the chemical composition of the products. Brunauer-Emmett-Teller (BET) specific surface area tests were carried out on a Thermo Fisher Surfer at 200 °C.

### Electrochemical Measurements

A typical three-electrode set-up was utilized for electrochemical measurement with CHI760D potentiostat (CH Instruments, China). All measurements of the HER activity were conducted using a 0.5 M H_2_SO_4_ (pH = 0.3) electrolyte after continuous purging with N_2_ gas. A glassy carbon electrode (GCE) with a diameter of 3 mm covered by a thin catalyst film was used as the working electrode. Typically, 6 mg catalyst was suspended in 1 ml water-ethanol mixed solution (volume ratio of 4:1) containing 20 μl Nafion solution to form a homogeneous ink assisted by ultrasonication. Then 20 ml of the ink was dropped onto the surface of glassy carbon by a micropipette and dried under room temperature. Saturated calomel electrode (SCE) and graphite rod (Pt anodes may dissolve in the electrolyte and contaminate the cathode) were used as reference electrode and counter electrode respectively. The electrocatalytic activities were examined by polarization curves using linear sweep voltammetry (LSV) at a scan rate of 5 mV/s with IR compensation, at room temperature. Before measurements, the samples were repeatedly swept from −0.5 to 0 V (versus SCE) in the electrolyte until a steady voltammogram curve was obtained. Potentials were referenced to a reversible hydrogen electrode (RHE) by adding a value of 0.262 V (0.244 + 0.0591 × pH).

## Results and Discussions

**Figure 1:**
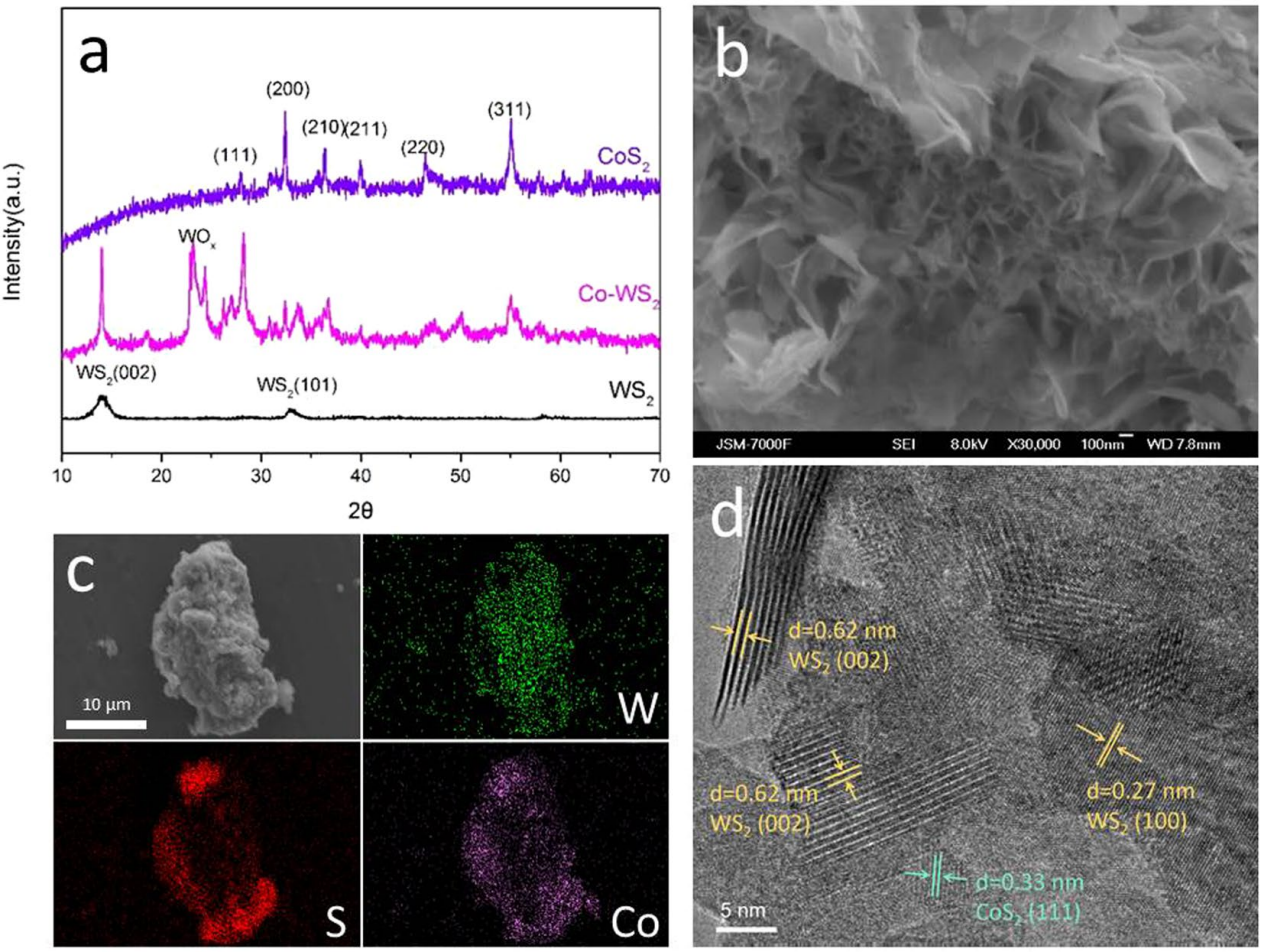
(**a**) XRD patterns of WS_2_, CoS_2_ and Co-doped WS_2_, (**b**) SEM image, (**c**) Element mapping images from EDS and (**d**) HRTEM image of Co-doped WS_2_.

The morphology and chemical composition of as-synthesized Co-WS_2_ are shown in Fig. 1. The XRD pattern in Fig. 1a shows the differences between Co-doped WS_2_ and pure CoS_2_. The diffraction peaks of both pure CoS_2_ and Co-doped WS_2_ correspond to CoS_2_ (JCPDS card no. 41–1471). However, the doped Co has a stronger (111) diffraction (27.9°), while pure CoS_2_ has stronger (200) and (311) diffractions, corresponding to peaks at 32.3° and 54.9° respectively. There are two peaks at 14.4° and 33.6°, assigned to diffractions of the (002) and (101) planes of WS_2_ (JCPDS card no. 08-0237) respectively. It is worth mentioning that the Co-doping also oxidizes WS_2_, with observed diffraction peaks at around 25°^26^. Though the mechanism of oxidation is still under investigation, tungsten oxides (WO_x_) have higher electrical conductivities than the metallic phase 1T-WS_2_, and may bring additional benefits to HER^27–29^. The typical three-dimensional nanosheet structures of WS_2_ can be observed in SEM (Fig. 1b), while massive self-nucleated CoS_2_ also exists (Fig. S2). EDS mapping (Fig. 1c) indicates a homogeneous distribution of Co element, instead of dissociative CoS_2_ shown in Fig. S2. Figure 1d shows a HRTEM image of Co-WS_2_, detailing the crystal structure of the product. The lattice plane (002) of WS_2_ with a spacing distance of about 6.2 Å can be observed, as well as the lattice plane (100) of WS_2_ with a shorter spacing distance of 2.7 Å. Another plane of middle spacing distance of 3.3 Å can be also found, which corresponds to the lattice plane (111) of CoS_2_, which corroborates with the XRD results.

**Figure 2:**
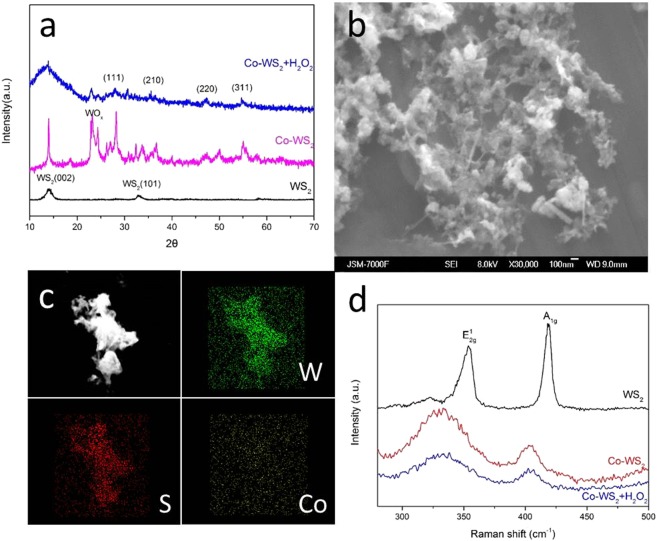
(**a**) XRD patterns of WS_2_, Co-doped WS_2_ and H_2_O_2_ treated Co-WS_2_, (**b**) SEM image and (**c**) Element mapping images from EDS of H_2_O_2_ treated Co-WS_2_, and (**d**) Raman spectra of WS_2_, Co-doped WS_2_ and H_2_O_2_ treated Co-WS_2_.

After etching of WS_2_ by H_2_O_2_, the intensity of CoS_2_ peaks decreased (Fig. 2a), corresponding to the notable decrease of Co content found in EDS mapping (Fig. 2c). However, the (002) diffraction peak of WS_2_ became broader, which is caused by poor crystallinity. The Co-WS_2_ samples became smaller after H_2_O_2_ etching and ultrasonication, while pieces of WS_2_ were still observed as displayed in Fig. 2b. Such a porous nanosheet structure is propitious for HER. Raman spectroscopy was conducted for WS_2_, Co-WS_2_ and H_2_O_2_ treated Co-WS_2_. As shown in Fig. 2d, the peaks observed at 353 cm^−1^ and 418 cm^−1^ correspond to the ${{\rm{E}}}_{2{\rm{g}}}^{1}$ and ${{\rm{A}}}_{1{\rm{g}}}$ modes of WS_2_, respectively. ${{\rm{E}}}_{2{\rm{g}}}^{1}$ is an in-plane optical mode, while ${{\rm{A}}}_{1{\rm{g}}}$ corresponds to out-of plane vibrations of the sulfur atoms^30,31^. However, both the two bands of Co-WS_2_ and H_2_O_2_ treated Co-WS_2_ show an obvious shift to 330 cm^−1^ and 404 cm^−1^ respectively, as compared to pure WS_2_. This change of red shift should be ascribed to the influence of small amounts of doping to the host materials^32^.

**Figure 3:**
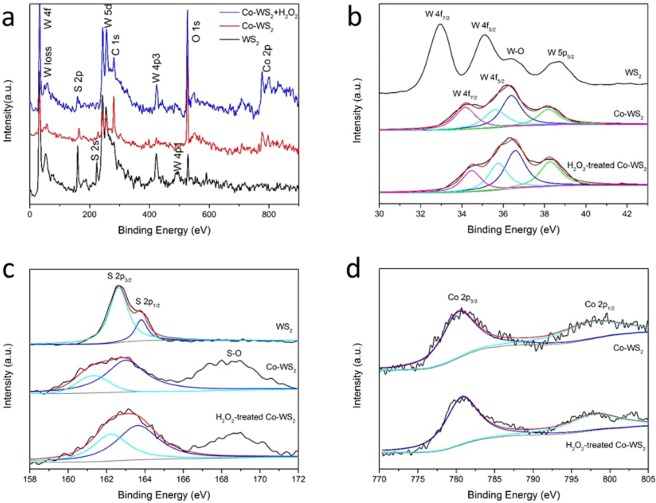
XPS spectra of WS_2_, Co-doped WS_2_ and H_2_O_2_ treated Co-WS_2_. (**a**) Survey scan, (**b**) W 4f, (**c**) S 2p and (**d**) Co 2p peaks, respectively.

X-ray photoelectron spectroscopy (XPS) spectra are shown in Fig. 3 to confirm the elemental composition of the products. Figure 3a shows the typical survey spectrum of WS_2_, Co-doped WS_2_ and H_2_O_2_ treated Co-WS_2_. Here, the presence of C 1s, W 4f, S 2p, and O 1 s regions may be observed in both samples. Specifically, Co 2p regions can be seen in Co-doped samples. In Fig. 3b, compared to bare WS_2_, two peaks of 4f-level W atoms shift to higher binding energy, which is probably caused by Co-doping. It is found that the W-O bond at 36.5 eV shows a relative increase in Co-doping samples, demonstrating oxidation along with Co-doping. Meanwhile, the conversion of W valence state from +4 (WS_2_) to +6 (WO_3_) could also result in the increase of binding energy^26^. In Fig. 3c, the S 2p_3/2_ and 2p_1/2_ peaks at 162.6 and 163.8 eV suggest the −2 valence state for S. Co-doping leads to the formation of S-O bond, shown as the peak at around 169 eV. This decreases the binding energy for S 2p bonds. Two peaks shown in Fig. 3d at binding energy of 780.6 and 798.0 eV corresponding to Co 2p_3/2_ and 2p_1/2_, are almost consistent with the results reported for CoS_2_^33^.

**Figure 4:**
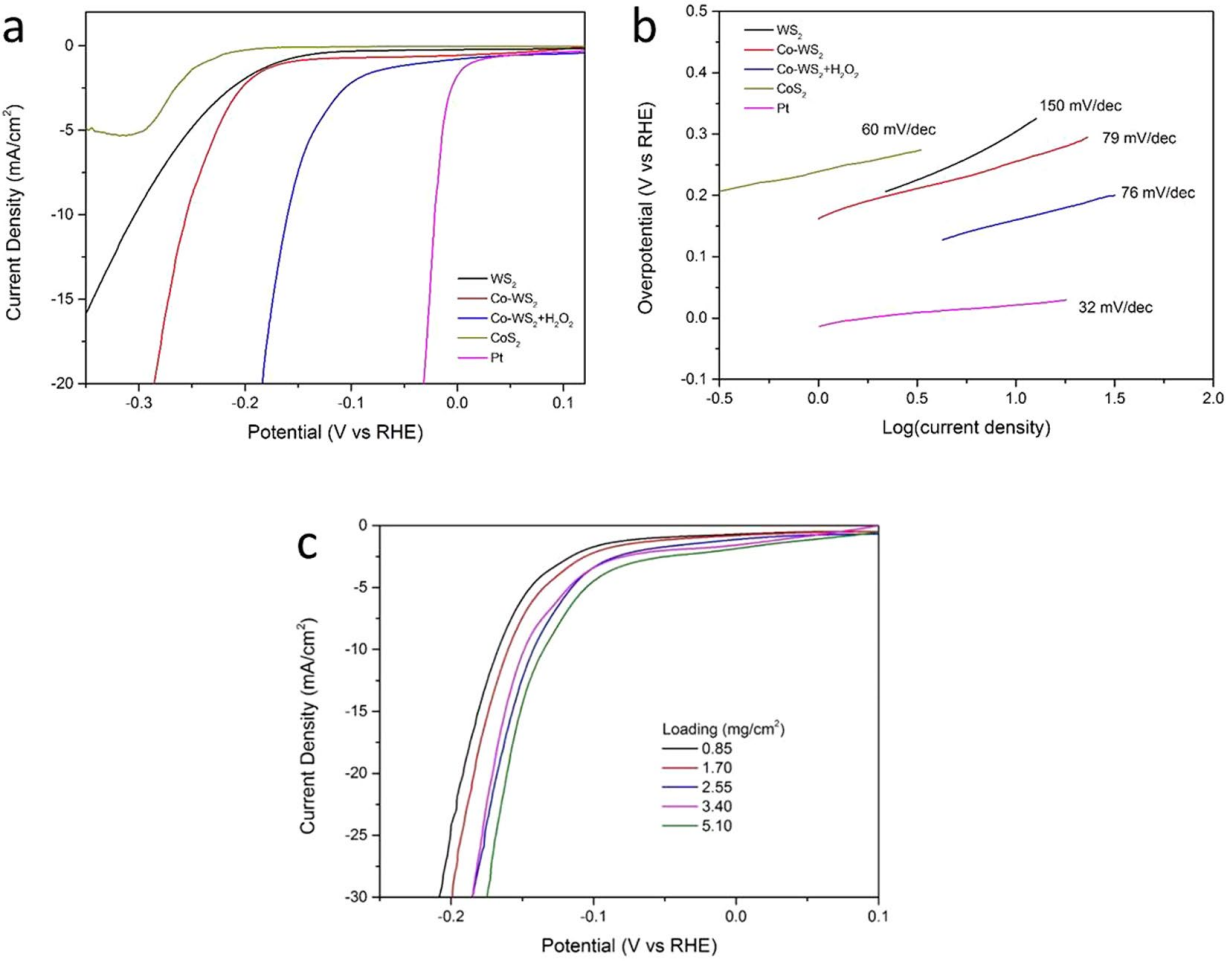
Effect of Co doping and H_2_O_2_ treatment on the HER (**a**) polarization curves and (**b**) corresponding Tafel plots of a series of samples loading of 1.7 mg/cm^2^. (**c**) Polarization curves recorded with different amount loading.

We tested the electrocatalytic HER performance using a simple three-electrode set-up in 0.5 M H_2_SO_4_ solution. Figure 4a shows the polarization curves measured by Linear-sweep voltammetry (LSV) at a scan rate 0.5 mV/s. Bare WS_2_ and bare CoS_2_ both showed poor HER performances, and CoS_2_ even showed a reduction peak during cathodic polarization that disappeared after hundreds of scans. The Co-doped WS_2_ sample showed a good HER performance with an overpotential of −255 mV at the current density of −10 mA/cm^2^. The influence of Co-doping with different amounts has been discussed, as shown in Fig. S3. Considering the self-nucleation of CoS_2_ at the surface of WS_2_, we have tried another method to realize Co-doping. The results are still under study, as displayed in Fig. S4.

The H_2_O_2_ treated Co-WS_2_ reached a much smaller overpotential of −160 mV at −10 mA/cm^2^, showing a better HER performance that approaches that of the commercial 20% Pt/C catalyst (−21 mV @ −10 mA/cm^2^). By fitting linear regions of the LSV plots to the Tafel equation η = a + b log j, where j is the current density and b is the Tafel slope^34^, the corresponding Tafel plots of each sample were shown in Fig. 4b. Tafel slopes of 20% Pt/C, H_2_O_2_-treated Co-WS_2_, Co-WS_2_, bare CoS_2_ and bare WS_2_ were extracted as 32, 76, 79, 60, and 150 mV/dec, respectively. The Co-doped WS_2_ samples showed smaller Tafel slopes than that of WS_2_, indicating a faster gain of HER velocity with increasing potential. Also, even though the HER activity of CoS_2_ is relatively low, the Tafel slope is similar to that of a Pt/C catalyst, which may reflect the effect of Co doping. Electrochemical impedance spectroscopy (EIS) was used to investigate the electrode kinetics under the HER test conditions, confirming the facile kinetics of H_2_O_2_-treated Co-WS_2_ toward HER (Fig. S5).

We then investigated the HER performance of the best sample (H_2_O_2_ treated Co-WS_2_) under different loadings, as shown in Fig. 4c. It was found that the catalytic performance got even better with heavier loading, and the overpotential could reach −134 mV when the loading on GCE was 5.1 mg/cm^2^. However, when the loading was further increased (by increasing the concentration of dispersion ink or enhancing the dropped amount on GCE), the dry film of catalyst on GCE became fragile and fell off easily, causing poor electrical conductivity and HER stability. Durability measurement of H_2_O_2_-treated Co-WS_2_ was carried out with a small loading of 0.85 mg/cm^2^ (Fig. S6).

Characterizations indicated that the Co-doping caused oxidation in the final products. Compared to bare WS_2_, Co-doped WS_2_ consists of W, Co, S, and O elements. We applied H_2_O_2_ in order to etch the surface of WS_2_, but also caused a massive loss of Co. Considering the better HER performance of H_2_O_2_ treated Co-WS_2_, we speculate that appropriate H_2_O_2_ etching could remove redundant self-nucleated CoS_2_ covering or mixed with WS_2_, which possesses poor HER activity. The remaining Co content plays a role in tuning the free energy of hydrogen adsorption of WS_2_ atoms, especially inert in-plane atoms. The above-mentioned lead to a highly efficient HER performance, manifested as good electrical conductivities of tungsten oxide (WO_x_).

**Figure 5:**
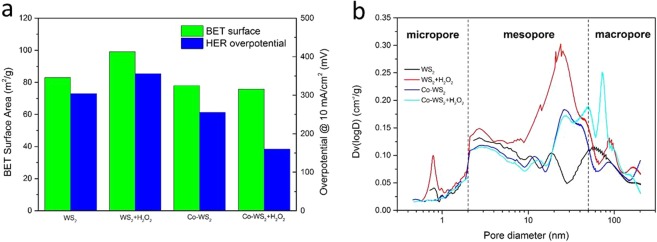
(**a**) Histogram of measured BET surface areas and HER overpotentials (at 10 mA/cm^2^), and (**b**) Pore size distributions based on BJH method of serial samples.

In order to quantitatively analyze the dependence of HER performance on sample microstructures, we carried out independent N_2_ adsorption measurements based on Brunauer-Emmett-Teller (BET) models on these powder samples. The BET surface area and HER overpotential (at 10 mA/cm^2^) of each sample were compared in Fig. 5a. Based on our understanding, bigger surface areas of catalyst should be associated with more active sites. Especially for WS_2_, the mesopores may increase the number of edges as the active sites and increase the accessibility of the catalyst surface. That is the reason why we chose H_2_O_2_ to etch WS_2_ into ultrathin and porous nanosheets. The BET measurement confirmed the influences of etching on WS_2_, and the surface area of WS_2_ was enhanced from 82.9 to 99.0 m^2^/g. The BET surface area of Co-doped WS_2_ is slightly smaller than that of WS_2_, due to the Co-doping into the interspace of WS_2_ and the self-nucleation of CoS_2_. However, the relation between HER performance and surface area of WS_2_ samples is unexpected. The higher surface area of H_2_O_2_-treated WS_2_ led to a worse HER performance than that of bare WS_2_, and the lowest surface area of H_2_O_2_-treated Co-WS_2_ corresponds to the best HER efficiency. For better understanding, pore size distributions were calculated from adsorption branches of isotherms by the Barrett-Joyner-Halenda (BJH) method, as shown in Fig. 5b. The detailed pore size distributions suggest that serial WS_2_ samples contain a mixture of macropores (>50 nm), mesopores (2–50 nm), and a small amount of micropores (<2 nm). Compared to bare WS_2_, the H_2_O_2_ etching caused an increase in mesopore distribution ranging from 10 to 50 nm, as well as micropores of 0.8 nm and macropores of 90 nm. The increased mesopores caused a larger BET surface area, but had no impact on the HER. The Co-doped WS_2_ also possesses a broad mesopore distribution, which is probably contributed by CoS_2_. The subsequent H_2_O_2_ treatment resulted in macropore distributions at 50 nm and 73 nm. The influence of H_2_O_2_ concentrations is discussed in supplementary materials (Fig. S7). Considering the above results, we speculate that both Co doping and H_2_O_2_ etching almost could not influence the pore (<10 nm) distribution of WS_2_. Thus the macropores etched by H_2_O_2_ associated with Co-doping may account for the enhanced HER performance.

**Figure 6:**
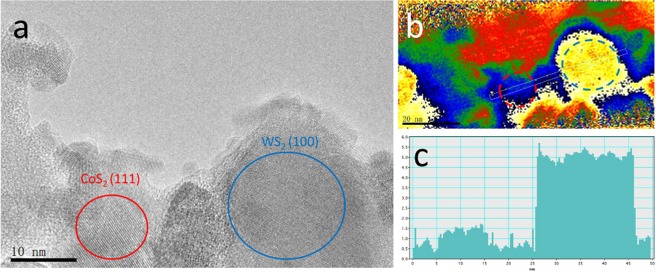
Electron holographic analysis: (**a**) The TEM image showing Co-doping nearby WS_2_, (**b**) phase reconstructed of the hologram corresponding to (**a**,**c**) phase profile measured along the strip in (**b**).

To understand the effect of doped Co content in the improvement of HER activity of WS_2_, electron holographic imaging has been taken to study the inner electrostatic potential of our samples. The amount of phase shift can provide a direct measure of the variation in electrostatic potential at the surface of nonmagnetic specimens with uniform thickness^35,36^. We selected a thin specimen containing CoS_2_ and WS_2_, as shown in Fig. 6(a). The corresponding hologram was taken using a transmission electron microscope equipped with an electron biprism and processed by two dimensional Fourier transformation to obtain the side-bands. The phase was reconstructed from the side-bands by inverse Fourier transformation, shown in Fig. 6(b). Figure 6(c) displayed the potential step along the dashed box in Fig. 6(b), showing a lower inner electrostatic potential in Co-doped regions of the sample.The Gibbs free energy of WS_2_ or MoS_2_ is too strong, leading to the poisoning of the active sites, especially in inner planes. Electronic states of in plane atoms would be significantly modulated by Co doping, thus leading to a moderate hydrogen adsorption free energy with a Gibbs free energy close to 0 for highly efficient HER^6,37^. The hologram may explain the modulation of electronic states. This is the first use of electrostatic potential measured by hologram to distinguish the activity in a single region of catalysts. DFT calculation is needed for further insights.

## Conclusions

In summary, we studied the influence of microstructure on high-performance Co-doped WS_2_-based HER catalyst. H_2_O_2_ etching was used to remove redundant self-nucleated CoS_2_ and modify the surface morphology of WS_2_, thus creating macropores. Various characterization results indicated that the products were composed of WS_2_, CoS_2_ and a spot of WO_x_, showing a low HER overpotential of −134 mV (10 mA/cm^2^ at a loading of 5.1 mg/cm^2^), with a small Tafel slope of 76 mV/dec. Co-doping associated with H_2_O_2_ resulted in an increasing macropore (>50 nm) distributions, which may account for the enhanced HER performance. For the first time, we used electron holographic analysis to study the electrostatic potential in Co-doped region. Further work is required, such as carbon material hybridization and DFT calculation, and we believe our study could bring in better understanding of promising catalysts design to replace noble metal for hydrogen production.

## Supplementary information

Multivariate Control of Effective Cobalt Doping in Tungsten Disulfide for Highly Efficient Hydrogen Evolution Reaction
