# Supplementary material for: Multivariate Control of Effective Cobalt Doping in Tungsten Disulfide for Highly Efficient Hydrogen Evolution Reaction
Source: Sci Rep. 2019 Feb 4;9:1357. doi: 10.1038/s41598-018-37598-0 (PMC6362019; doi:10.1038/s41598-018-37598-0)
Supplement: Supplementary file 1 — Multivariate Control of Effective Cobalt Doping in Tungsten Disulfide for Highly Efficient Hydrogen Evolution Reaction [file 41598_2018_37598_MOESM1_ESM.pdf]

Multivariate Control of Effective Cobalt Doping in Tungsten Disulfide for Highly Efficient Hydrogen Evolution Reaction

Liyan Zhou[1, 3], Shancheng Yan[2,], Haizeng Song[2], Han Wu[1, 3], Yi Shi[1, 3]

[1]Collaborative Innovation Center of Advanced Microstructures, Nanjing University, Nanjing 210093, P. R. China

[2]School of Geography and Biological Information, Nanjing University of Posts and Telecommunications, Nanjing 210023, P. R. China

[3]National Laboratory of Solid State Microstructures, School of Electronic Science and Engineering, Nanjing University, Nanjing 210093, P. R. China

Correspondence and requests for materials should be addressed to S.C.Y (email: yansc@njupt.edu.cn) or Y.S (email: yshi@nju.edu.cn)

Tel: +86 25-85866634; Fax: +86 25-85866634

Supplementary Material

4-nitrobenzene-diazonium (4-NBD) (Sigma-Aldrich) is commonly used to modify carbon materials (carbon nanotubes, graphene) and even black phosphorus (BP) [1], owning to its covalent carbon bonds at surface. Here, this aryl diazonium chemistry is tried as a route for the surface functionalization of WS_2 for HER stability. The related HER polarization curves were shown in **Fig. S1a**. However, it was found that this treatment could not help enhance the HER performance, and even may cause the passivation of active sites at WS_2 surfaces. Fortunately, we found that the annealing condition may significantly influence the catalytic activity of WS_2, thus series of comparative tests were carried out as shown in **Fig. S1b.** High temperature annealing may help expose the active sites and enhance the stability of surface structure [2]. Finally, bare WS_2 sample reached -304 mV at 10 mA/cm^2 when annealed under 450℃ for 4 hours. This annealing condition was chosen to be a necessary step during this work.

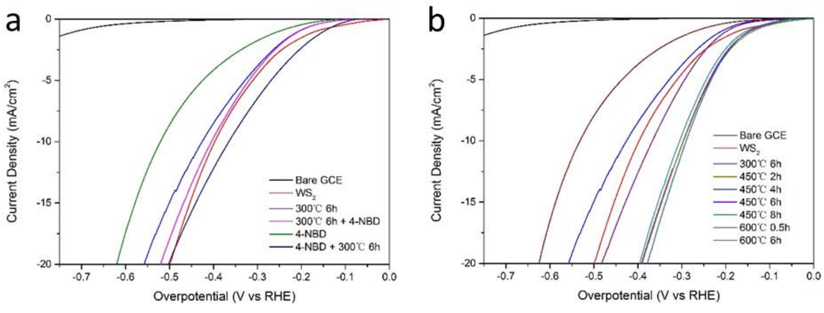

Figure S1. HER polarization curves of WS_2 samples under different (a) passivation by 4-NBD and (b) annealing conditions.

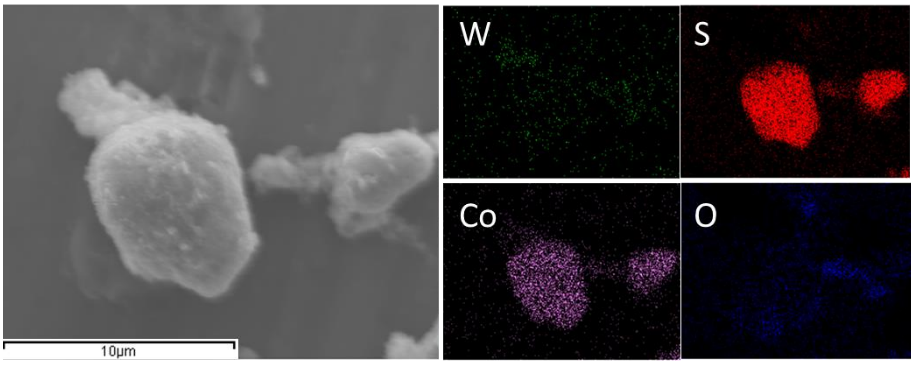

Figure S2. SEM image and Element mapping images of CoS_2.

We carried out control experiments to determine the suitable amount of Co-doping. The compositional variation of WS_2 samples with different Co-doping contents is shown in **Fig. S3a**. Along with the increase of Co-doping, the (111) diffraction peak (27.9 °) becomes stronger as well as the oxidation peak at around 25 °. The HER activity is also enhanced when Co-doping is increased, until a theoretical 23 wt% of Co in WS_2. Beyond this ratio, then the overpotential of sample became worse, probably due to the over nucleation of CoS_2 and distinct change in surface structure. Therefore, 23 wt% is chosen to be the best Co-doping ratio.

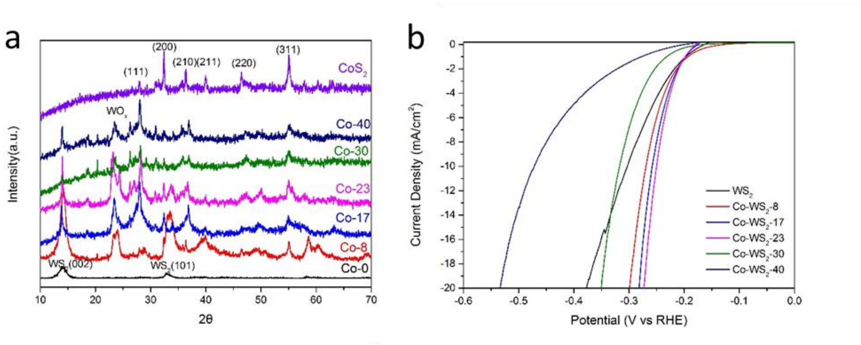

Figure S3. (a) XRD and (b) HER polarization curves of WS_2 samples with different Co-doping contents.

It was reported that low concentration of the Co thiourea complex $\mathrm{Co(thiourea)}_4^{2+}$ could refill some of the surface sulfur vacancy sites of MoS_2, instead of inducing a fast self-nucleation (as shown in **Fig. S2**) [3]. We employed this method as a control for Co-doping. 50.6 mg of $Co(NO_3)_2$ $6H_2O$ and 36 mg of thiourea (99%, Shanghai Lingfeng Chemical Regent Co. Ltd.) were added to 1 ml of water and left overnight to form the Co thiourea complex $\mathrm{Co(thiourea)}_4^{2+}$. 100 mg of as-prepared H_2O_2-treated WS_2 and 50 mg of polyvinylpyrrolidone (PVP, 99.9%, Sinopharm Chemical Reagent Co. Ltd.) were dispersed in 30 ml of 30 v/v% isopropanol (99.7%, Sinopharm Chemical Reagent Co. Ltd.)/water to form a mixed homogeneous solution, and different amount (1 to 9 ml) of this Co thiourea complex solution was added. Then the solution was transferred to an autoclave, followed by hydrothermal treatment at 160 ℃ for 24 h. After cooling to room temperature, the products were centrifuged and washed several times with DI water and ethyl alcohol, and dried at 60 ℃. This method doped Co in the form of CoS, with a strong (100) diffraction at 30.6° (JCPDS card no. 65-3418), shown in **Fig. S4a**. WS_2 samples doped in this method showed lower overpotentials when the added amount of Co thiourea complex was increased. However, the lowest HER overpotential (-224 mV at 10 mA/cm^2, loading 1.7 mg/cm^2) is still mediocre, shown in **Fig. S4b**.

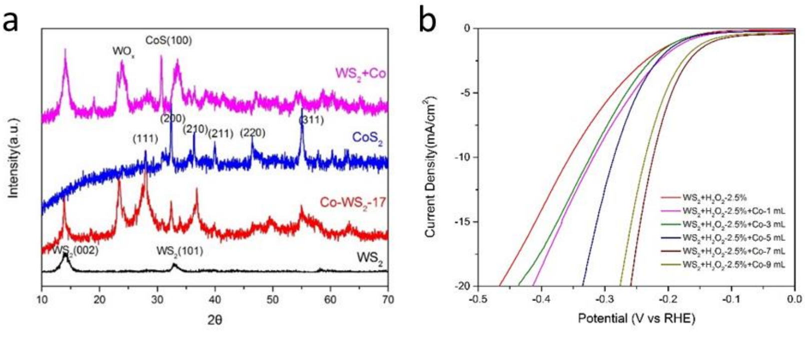

Figure S4. (a) XRD of WS_2 samples doped by Co thiourea complex and (b) HER polarization curves of serial Co thiourea complex doped WS_2.

Nyquist plots (**Fig. S5**) reveal dramatically reduced charge transfer resistances for the Co-doped WS_2 samples, compared to bare WS_2 (WS_2 > Co-WS_2 > H_2O_2-treated Co-WS_2). The EIS data support that H_2O_2-treated Co-WS_2 owns facile kinetics toward HER, corresponding to the best performance.

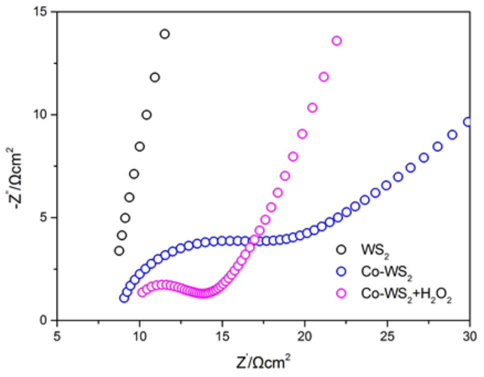

Figure S5. EIS Nyquist plots of WS_2, Co-WS_2 and H_2O_2-treated Co-WS_2.

Cyclic voltammetry curves of WS_2, Co-doped WS_2 and H_2O_2-treated Co-WS_2 at different scan rates and the calculated double-layer capacitance (C_{dl}) of corresponding catalysts are summarized in **Fig. S6**. Co doping slightly increased the capacitance of WS_2 (from 1.03 to 1.31 mF/cm^2), along with the enhancement of HER performance, while H_2O_2 treatment brought in a decrease of capacitance.

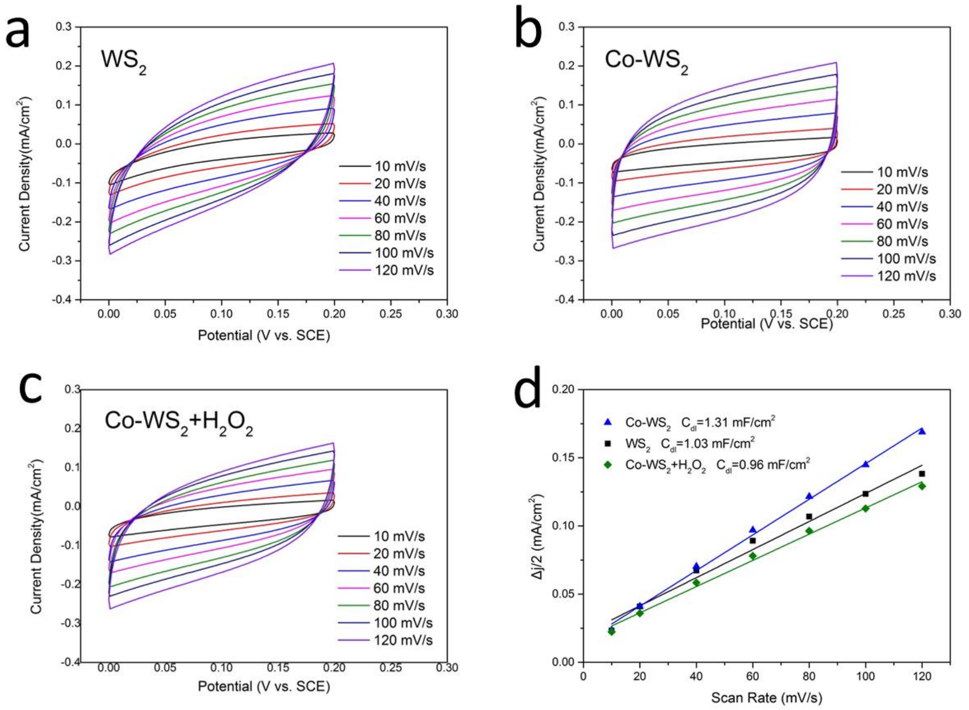

Figure S6. Cyclic voltammetry curves of (a)WS_2, (b)Co-doped WS_2 and (c)H_2O_2-treated Co-WS_2 at different scan rates ranging from 10 mV/s to 120 mV/s, and (d)the double-layer capacitance (C_{dl}) calculated by liner fitting of corresponding catalysts.

Durability measurements of H_2O_2-treated Co-WS_2 were carried out with a small loading of 0.85 mg/cm^2 (**Fig. S7**). There was a slightly increase of HER overpotential (about 20 mV) after 100 cycles of LSV, and another 20 mV of overpotential increase after 1000 cycles. This decline of HER performance may be ascribed to the slight fragmentation of catalyst film on GCE during the releasing of hydrogen bubbles.

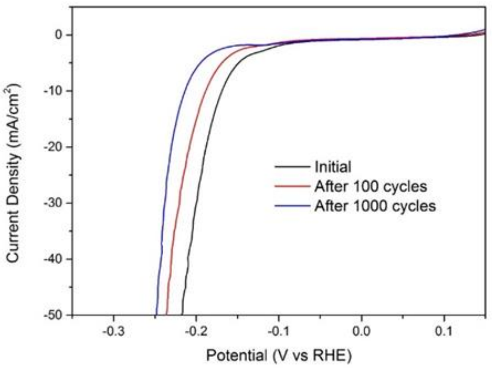

Figure S7. Durability measurement of H_2O_2-treated Co-WS_2.

Control experiments of the H_2O_2 concentrations were also carried out. It was observed that the suspension color of H_2O_2-treated Co-doped WS_2 was lavender, and the color got darker when using higher concentration of H_2O_2. There is no obvious change in BET surface area of Co-doped samples treated by H_2O_2 of different concentrations (**Fig. S8a**). But the etching actually brought in more pore distributions, especially macropores (**Fig. S8b**). 2.5 vol% was chosen to be the applied concentration of H_2O_2 etching, with the most macropore distribution and best HER performance.

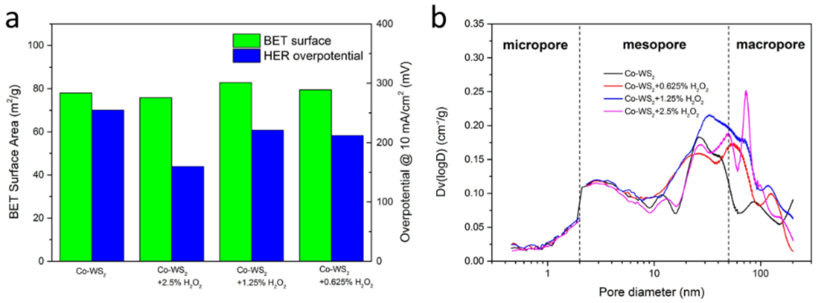

Figure S8. (a) Histogram of measured BET surface areas and HER overpotentials (@10 mA/cm^2), and (b) Pore size distributions based on BJH method of Co-doped WS_2 and etched samples by H_2O_2 of different concentrations.

The phase shift of hologram can provide a direct measure of the variation in electrostatic potential as well as thickness. In single WS_2 region (**Fig. S9a**), the phase shifts along with the

variation of thickness, as shown in **Fig. S9b**.

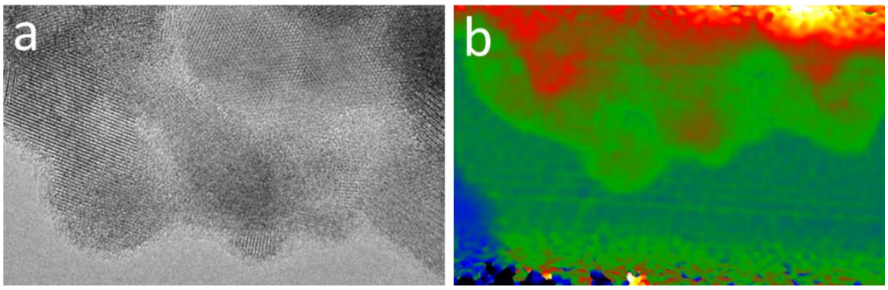

Figure S9. (a) The TEM image showing WS_2 edge and (b) phase reconstructed of the corresponding electron hologram.

References

1 Ryder, C. R. *et al.* Covalent functionalization and passivation of exfoliated black phosphorus via aryl diazonium chemistry. *Nat. Chem.* **8**, 597-602, doi:10.1038/nchem.2505 (2016).

2 Kiriya, D. *et al.* General Thermal Texturization Process of MoS2 for Efficient Electrocatalytic Hydrogen Evolution Reaction. *Nano Lett.* **16**, 4047-4053, doi:10.1021/acs.nanolett.6b00569 (2016).

3 Liu, G. *et al.* MoS2 monolayer catalyst doped with isolated Co atoms for the hydrodeoxygenation reaction. *Nat. Chem.* **9**, 810-816, doi:10.1038/nchem.2740 (2017).
